# Cyclic Generative Attention-Adversarial Network for Low-Light Image Enhancement

**DOI:** 10.3390/s23156990

**Published:** 2023-08-07

**Authors:** Tong Zhen, Daxin Peng, Zhihui Li

**Affiliations:** 1 College of Information Science and Engineering, Henan University of Technology, Zhengzhou 450001, China; zt@haut.edu.cn (T.Z.); peak-adam@stu.haut.edu.cn (D.P.); 2 Key Laboratory of Grain Information Processing and Control, Ministry of Education, Henan University of Technology, Zhengzhou 450001, China

**Keywords:** image enhancement in low-light conditions, generative adversarial networks, attention mechanisms, unsupervised learning

## Abstract

Images captured under complex conditions frequently have low quality, and image performance obtained under low-light conditions is poor and does not satisfy subsequent engineering processing. The goal of low-light image enhancement is to restore low-light images to normal illumination levels. Although many methods have emerged in this field, they are inadequate for dealing with noise, color deviation, and exposure issues. To address these issues, we present CGAAN, a new unsupervised generative adversarial network that combines a new attention module and a new normalization function based on cycle generative adversarial networks and employs a global–local discriminator trained with unpaired low-light and normal-light images and stylized region loss. Our attention generates feature maps via global and average pooling, and the weights of different feature maps are calculated by multiplying learnable parameters and feature maps in the appropriate order. These weights indicate the significance of corresponding features. Specifically, our attention is a feature map attention mechanism that improves the network’s feature-extraction ability by distinguishing the normal light domain from the low-light domain to obtain an attention map to solve the color bias and exposure problems. The style region loss guides the network to more effectively eliminate the effects of noise. The new normalization function we present preserves more semantic information while normalizing the image, which can guide the model to recover more details and improve image quality even further. The experimental results demonstrate that the proposed method can produce good results that are useful for practical applications.

## 1. Introduction

Images captured under complex conditions and with equipment limitations often have extremely poor quality due to inadequate illumination, resulting in image detail degradation, color distortion, and the presence of severe noise. These issues have a significant impact on subsequent vision tasks such as target object detection, semantic segmentation, and so on. To address these issues, low-light image enhancement has emerged as a critical task in image processing. Low-light image enhancement can improve the image’s visual quality and restore the image’s detailed information, which is useful for subsequent visual tasks.

The two methods listed below can be used to improve image quality. The first is to improve the hardware performance of image acquisition equipment, and the second is to process the collected images. The first method, on the other hand, has the disadvantages of high cost, difficult fabrication, and complex technology, as well as being relatively difficult to implement. As a result, using the improved algorithm to process the collected low-light images makes more sense.

Traditional low-light image-enhancement methods and deep learning-based low-light image-enhancement methods are the two types of low-light image-enhancement methods. Traditional methods for improving image quality were predominantly used in the early research on low-light image enhancement. Traditional low-light image-enhancement methods are classified as histogram equalization methods and Retinex theory methods. The histogram equalization method primarily works on the image’s pixels, ensuring that the pixels are evenly distributed across the entire image domain. Many methods have been proposed as research has progressed. A double histogram-equalization enhancement algorithm was proposed by Paul et al. [1]. The image’s histogram is modified by power-logarithmic transformation and then divided into two histograms for processing in this method, which effectively improves the image’s quality. However, some detailed information may be lost using this method. In response to this issue, Paul A. proposed a three-sub-histogram enhancement method for the three-platform limit [2]. Obtaining the average and median values from each sub-histogram effectively preserves the image’s detailed information. Although the above method improves image quality, it ignores the neighborhood relationship between pixels, resulting in noise in the enhanced image. To address this issue, Agrawal et al. [3] proposed a new method of joint histogram equalization. This method makes full use of the relationship between each pixel and adjacent pixels to effectively improve image quality, but it also introduces unnecessary artifacts and color distortion while enhancing. To address the aforementioned issues, Jebadass et al. proposed histogram equalization of intuitionistic fuzzy sets [4], and Mayathevar et al. used the weighted distribution algorithm to equalize the fuzzy color histogram [5]. Although the above method produced good results, the effect for different exposure areas is still not ideal. Tan proposed multiple histogram equalization (ERMHE) based on this [6], but there are issues, such as enhancement artifacts. To address the aforementioned issues, histogram equalization of three-way sub-images [7] and four-dimensional dynamic histogram equalization with limited contrast [8] are proposed. Although the above method’s proposal achieved some effects, it did not completely solve the problems of color cast, overexposure, and so on, and the quality of the enhanced image obtained is still not very good. Wang et al. [9] combined Retinex theory with the Gabor filter to improve images in the HSI and RGB color spaces as their research progressed. Over-enhancement, halos, and insufficient detail preservation have all been addressed. To produce better visual effects while preserving image details, Chen et al. [10] improved the Retinex method with a fully variational model and an adaptive gamma transform. The authors of [11] divided the input image into reflective and illuminated components using the Retinex theory and added edge preservation to the illuminated component. The methods described above solve specific problems to varying degrees, but they also introduce artifacts and noise. The traditional method focuses primarily on the relationship between the pixels of the image itself, but this method does not account for factors such as noise, making it difficult to achieve the ideal enhancement effect. In recent years, the widespread use of deep learning in computer vision has led to its application in improving low-light images. The majority of existing low-light image-enhancement methods rely on paired datasets to complete low-light image-enhancement tasks. In the literature, a progressive recursive image-enhancement network (PRIEN) has been proposed [12]. Its primary goal is to extract features iteratively through the use of recursive modules composed of recursive layers and residual blocks. According to the literature [13], an end-to-end enhancement network is built with a module stacking method and attention blocks, and the image is then enhanced with fusion. Because most methods focus on enhancing image luminance while ignoring image detail information, the literature [14] proposes the DELLIE algorithm, which focuses on image detail information extraction and fusion, thus recovering image detail information while maximizing image semantic information retention. However, the method described above does not achieve a balance of light intensity, color retention, and detail information. Liu et al. [15] proposed a local adaptive embedding network to solve this problem. This method achieves low-light image enhancement by utilizing adaptive feature selection and attention that can perceive global and local details. But this supervised approach relies on paired datasets, which are often difficult to obtain in practice. Based on this, researchers attempted to improve low-light images using an alternative, unsupervised approach. Kandula et al. [16] proposed a low-light image-enhancement method for adaptive lighting. This method addresses the issue of insufficient enhancement under various lighting conditions by introducing an illumination adaptive-enhancement network. EnlightenGAN [17] improves low-light images using an unsupervised approach that can be trained on unpaired datasets and employs a global–local discriminator structure as well as a self-constrained perceptual loss. However, the noise is still difficult to eliminate. To address the issue of noise, MAGAN [18] improves low-light images by introducing a hybrid attention module layer that models the relationship between each pixel in an image and its surroundings. The relationship between features is modeled to improve low-light images. The methods described above are all aimed at underexposure, and the image-processing effect on overexposure is relatively poor. On the basis of this, Nguyen et al. [19] proposed a progressive low-light image-enhancement network. This method effectively addresses the image’s exposure issue. Furthermore, in some cases, these methods are unable to remove the effects of color bias, artifacts, and noise. This clearly affects the quality of the image.

**Figure 1:**
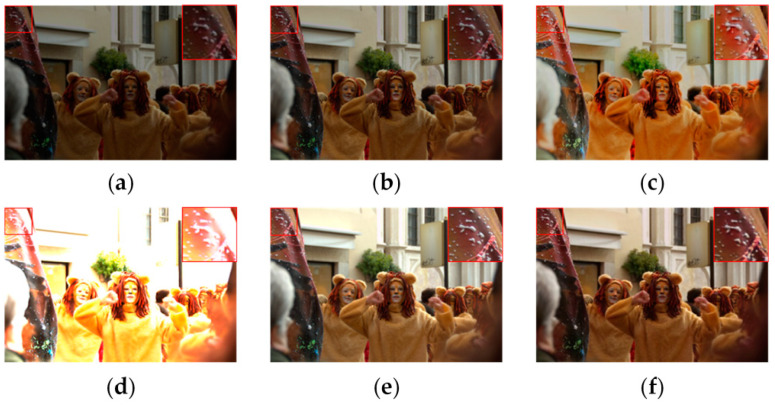
Comparison of our method with other methods after enhancement. Our method preserves color information and is closest to the real image in the area denoted by red boxes, yielding good results. (**a**) Input, (**b**) DALE, (**c**) DRBN, (**d**) RUAS, (**e**) Ground Truth, (**f**) ours.

To address the issues raised above, we propose a cyclic generative adversarial attention network (CGAAN). The network is completely unsupervised and can maximize the conversion of low-light images to normal-light images without the use of paired datasets. Figure 1 depicts a comparison of our method to other methods. As shown in Figure 1, the areas highlighted in red are significantly enhanced, and unlike other methods, they do not introduce color bias. It is clear that our method is more accurate. In order to better improve the image quality, we introduce a new attention mechanism, namely adaptive feature attention. This attention mechanism differs from other attention mechanisms that encode contextual information in that global average pooling and maximum pooling operations are performed on the encoded feature maps obtained by downsampling and residual block processing, and the parameters of each channel are combined to assign weight parameters, enabling an adaptive attention mechanism under the feature maps. This attention mechanism guides the network to better recover high-quality images by focusing on the important regions in low-light images. Also, the introduction of region loss enables the network to better distinguish the low-light domain from the normal-light domain to extract more important features, which helps improve the network’s ability to recover detailed information. We use adaptive instance layer regularization based on cyclic generative adversarial networks to eliminate color bias to some extent, allowing the network to retain more texture structure and learn the global correlation between channels.

In summary, the main contributions of our paper are as follows:

(1) We present CGAAN, an unsupervised low-light image-enhancement method that has been demonstrated experimentally to perform well;
(2) Based on the cyclic generative adversarial network, we present a novel attention mechanism. This attention mechanism can direct the network to enhance different regions to varying degrees, depending on whether they are in low or normal light;
(3) To make the generated images more realistic, we add a stylized region loss function and a new regularization function on top of the cyclic adversarial network.

## 2. Related Work

This section provides a brief overview of the current state of research in low-light image enhancement, including both traditional and deep learning methods. This is followed by a review of advances in attention mechanisms and, finally, a review of methods for generating adversarial networks. Table 1 shows a comparison of our method to existing methods.

### 2.1. Traditional Methods

Histogram equalization methods and Retinex theory methods are the main and most widely used methods in the field of low-light image enhancement. The histogram equalization method focuses on the image’s pixels, primarily to ensure that the pixels are evenly distributed across the image domain. This method, however, has some limitations and frequently results in the loss of contextual details, severe chromatic aberration, and some noise. Many algorithms have been proposed to solve these problems. For example, histogram equalization (HE) [20], but this method has the limitation of not taking into account each pixel’s neighborhood information. Following that, Agrawal et al. [3] proposed a new method for joint-histogram equalization. This method truly considers the image’s two-dimensional information and improves the image’s contrast. Many new methods were proposed as technology matured, such as histogram equalization with intuitionistic blur sets [4], equalization of blurred color histograms using a weighted distribution algorithm [5], exposure region-based multiple-histogram equalization (ERMHE) [6], histogram equalization of tri-square sub-images [7], and four-dimensional dynamic histogram equalization with limited contrast [8]. However, these methods do not completely solve the color shift and noise artifact problems in low-light image enhancement in low-light conditions. Retinex-based methods for image enhancement were proposed, which decompose the image into illuminated and reflected images and remove the effect of the illuminated image. Among the Retinex methods, single-scale Retinex [21] and multi-scale Retinex [22] are pioneering studies that use the reflected component as the final output. Wang et al. [9] used the Gabor filter in conjunction with Retinex theory to improve images in both the HSI and RGB color spaces. Insufficient detail retention, halation, and over-enhancement were all addressed. Chen et al. [10] enhanced the Retinex method with a full variational model and an adaptive gamma transform to produce better visual effects while preserving image details. Lin et al. [11] used the Retinex theory to divide the input image into reflection and illumination components, and they added edge-holding to the illumination component. Yang et al. [23] used Retinex theory in conjunction with a fast and robust fuzzy C-mean clustering algorithm to estimate the initial illumination image and then performed segmentation and fusion to enrich the image’s details. However, due to the lack of a reflectance constraint, these methods tend to amplify potential dense noise or even artifacts in low-illumination images.

**Table 1:** Comparison of existing image-enhancement methods and our method in low-light conditions.

Method	Advantage	Disadvantage
EnlightenGAN [17]	A global–local discriminator is being introduced. Overfitting was eliminated, and the model’s generalization ability was enhanced.	Noise is difficult to eliminate.
MAGAN [18]	Improve low-light images and remove potential noise by using a hybrid attention layer to model the relationship between each pixel and the image’s features.	When the finished product is obtained, pixel-by-pixel addition has some limitations.
HE [20]	Increase the image’s contrast.	Each pixel’s neighborhood information is ignored.
Agrawal [3]	This method fully utilizes the relationship between each pixel and its neighbors, resulting in improved image quality.	Enhancements can also cause artifacts and color distortion.
Literature [4]	Artifacts and color distortion issues have been addressed to some extent.	The processing effect for various exposure areas is still not optimal.
ERMHE [6]	Multi-histogram equalization improves the contrast of images that are not uniformly illuminated.	There are some issues, such as enhancement artifacts.
Literature [7]	Divide the image into three histograms and equalize each one separately.	There could be over-enhancement.
Literature [8]	The use of four-histogram equalization with limited contrast compensates for the flaws of over-enhancing and over-smoothing.	Does not fully address color shift and noise artifacts in image enhancement in low-light conditions.
Literature [21]	As the final output, use the reflection component.	Inadequate detail preservation, halos, and over-enhancement issues.
Wang [9]	Using Gabor filters in conjunction with Retinex theory to enhance images in the HSI and RGB color spaces.	The image does not appear to be natural.
Chen [10]	The Retinex method is improved with a fully variational model and an adaptive gamma transformation to produce better visuals while preserving image details.	Artifacts could be present.
Lin [11]	Using Retinex theory, divide the input image into reflection and illumination components and then add edge preservation to the illumination component.	Will generate noise.
Yang [23]	To estimate an initial illuminated image, Retinex theory was combined with a fast and robust fuzzy C-means clustering algorithm, followed by segmentation and fusion to enrich the image details.	Inadequate constraints on reflection components, potentially introducing artifacts and noise.
LACN [24]	Introduce a parameter-free attention module and propose a new attention module that retains color information while improving brightness and contrast.	Does not take into account global information.
PRIEN [12]	A recurrent module composed of recurrent layers and residual blocks is used to extract features iteratively.	The image’s details are ignored.
Literature [13]	Create an end-to-end augmentation network using a module stacking approach and attention blocks, then use fusion to augment images.	The image’s details are ignored.
DELLIE [14]	When combined with the detail component prediction model, it is possible to extract and fuse image detail features.	Unable to strike a balance between lighting and detail information.
Liu [15]	Image enhancement in low-light conditions using adaptive feature selection and attention that can perceive global and local details.	Inadequate enhancement in various lighting conditions.
Kandula [16]	Enhance images in two stages with a context-guided adaptive canonical unsupervised enhancement network.	The image’s texture and semantic information are unaffected.
FLA-Net [25]	Using the LBP module, concentrate on the image’s texture information.	There will be issues with color distortion.
Literature [26]	To address the issue of color distortion, use a structured texture-aware network and a color-loss function.	Maintaining a natural image is difficult.
Retinex-Net [27]	Used in conjunction with Retinex theory to adjust lighting components.	The image’s specifics are ignored.
R2RNet [28]	Enhance images using three sub-networks and frequency information to retain details.	Inability to adapt.
URetinex-Net [29]	The decomposition problem is formulated as an implicit prior regularization problem for adaptive enhancement of low-light images.	When something is inefficient, there will be some distortion.
Zero-DCE [30]	Deep networks are used to transform image augmentation into image-specific curve estimation.	Noise suppression is ineffective.
Literature [31]	Image enhancement with two-stage light enhancement and noise-suppression networks.	Image quality could be improved.
Proposed method	The addition of feature attention, style area loss, and adaptive normalization functions improves image quality.	The runtime may be extended due to network design.

### 2.2. Deep Learning Methods

Deep learning has significantly impacted the field of image processing in recent years and has a wide range of applications. This section will discuss some deep learning-related low-light image-enhancement methods. These methods are classified as supervised or unsupervised.

Supervised methods. The supervised method requires the use of labeled training data. The majority of research on low-light image enhancement has been done by building end-to-end network models based on paired datasets. The literature [24] proposes a lightweight attention-guided ConvNeXt network (LACN) for low-light image enhancement, which introduces parameter-free attention modules and proposes a novel attention module for building a lightweight network that preserves color information while enhancing brightness and contrast. To achieve low-light image enhancement, a progressive recursive image-enhancement network (PRIEN) is proposed in the literature [12]. Its primary goal is to extract features iteratively through the use of a recursive module composed of recursive layers and residual blocks. In the literature [13], an end-to-end enhancement network was built using a module stacking approach and attention blocks, and then the image was enhanced using fusion. Because most methods focus on enhancing image brightness while ignoring image detail information, the literature 14 proposed the DELLIE algorithm, which focuses on the extraction and fusion of image detail information, thereby recovering image detail information. Liu et al. [15] proposed a local adaptive embedding network. This method achieves low-light image enhancement by utilizing adaptive feature selection and attention that can perceive global and local details. Kandula et al. [16] proposed a low-light image-enhancement method for adaptive lighting. This method addresses the issue of insufficient enhancement under various lighting conditions by introducing an illumination adaptive-enhancement network. To improve the image enhancement effect, the literature [25] proposed a multi-stage modular network (FLA-Net) that focuses more on the texture information of the image via the LBP module. The literature [26] proposed a structural texture-aware network that solves the color distortion problem using a color-loss function. A popular direction is to combine the supervised approach with the Retinex theory. Retinex-Net [27] decomposes the input image into reflection and illumination components and then adjusts the illumination components using an encoder–decoder network. R2RNet was proposed in the literature [28], which uses three sub-networks of decomposition, denoising, and enhancement to enhance the image while retaining details. According to the literature [29], a Retinex-based depth-unfolding network (URetinex-Net) decomposes low-light images into reflection and illumination blocks and adaptively enhances low-light images by formulating the decomposition problem as an implicit priori regularization. The method described above is highly dependent on the dataset and could be improved in real-world scenarios.

Unsupervised methods. Unlike supervised methods, unsupervised methods do not require paired datasets but directly learn the rules and hidden features from the data itself, which greatly improves the efficiency of the processing. In the literature [30], a zero-reference depth curve estimation (Zero-DCE) method was proposed, which reduces image enhancement to image-specific curve estimation using a deep network. The EnlightenGAN method was proposed in the literature [17], which creates a new global–local discriminator as well as a new self-normative perceptual loss that fuses attention mechanisms to improve images. The literature [31] describes a GAN-based two-stage enhancement network that enhances images with two stages of light enhancement and noise suppression. The literature [18] proposed MAGAN, which improves low-light images and removes potential noise by introducing a hybrid attention layer to model the relationship between each pixel and feature in the image. Although the preceding method does not require a paired dataset, it still does not deal well with noise and exposure issues.

### 2.3. Attention Mechanism

Traditional networks often struggle to capture important regions of objects in low-light conditions. As a result, researchers proposed an attention mechanism [32], which is used to improve the network’s attention to important information and, thus, its ability to extract features. By assigning different weight coefficients to different areas, the attention mechanism achieves the goal of focusing on the target area. There are two types of attention: spatial attention and channel attention. Jie proposed the SE attention mechanism [33], which takes into account the relationship between feature channels and adds an attention mechanism to them. It learns the weights of each channel in order to focus on important features and suppress unimportant features, thereby improving the network’s feature expression ability. Further research discovered that channel features alone are insufficient to improve the network’s feature-extraction ability. Woo [34] proposed the CBAM attention mechanism, which combines the two dimensions of feature channel and feature space. The attention mechanism integrates the important features of two dimensions, pays more attention to the image’s detailed information, and improves the network’s feature expression ability. Wang [35] proposed ECA, a new and efficient channel-focused attention mechanism. It can improve information extraction and performance with a small number of parameters. Hou [36] proposed a new, efficient attention mechanism. It takes location information into account and prioritizes global information. The attention mechanism has been significantly improved in recent years [37,38,39,40,41], which has the advantage of paying more attention to target regions. With its distinct advantages, the attention mechanism has been applied to a wide range of computer vision fields.

### 2.4. Generative Adversarial Networks

In recent years, generative adversarial networks [42] have seen widespread use in computer vision. The original generative adversarial network consists of two networks: the generative network and the discriminative network. To trick the discriminative network, the generative network generates real synthetic samples from the noise distribution. The discriminative network’s goal is to distinguish between genuine and false samples. In addition to random samples from noisy distributions, the generator can accept various types of data as input. Generative adversarial networks have been widely used to remove motion blur from images [43], as well as noise [44]. With further research, GAN shines in the field of image generation and has derived many methods, CycleGAN [45] being one of them. CycleGAN has been used successfully in image enhancement, image denoising, image deblurring, and other fields. CycleGAN takes unpaired images as input and generates more realistic images than other unpaired image methods. The double generator and discriminator structure can ensure that the generated target image contains the same semantic information and can maximize the quality of the generated image. We use the CycleGAN framework in the proposed model because of the advantages listed above.

## 3. Proposed Method

Traditional GAN-based image-enhancement methods require paired data and input images, which can be difficult to obtain in practice. Traditional GAN methods, on the other hand, have few constraints and cannot guarantee the integrity of semantic information. Because of the aforementioned issues, the quality of target images generated by traditional GAN methods is frequently low. Therefore, we utilized the CycleGAN framework to generate high-quality images.

U-Net [46] has achieved great success in semantic segmentation, image restoration, and enhancement with additional research [47]. By extracting multi-level features from network layers of varying depths, U-Net preserves rich texture information and synthesizes high-quality images using multi-scale contextual information. U-Net has a wide range of applications in many fields due to its unique advantages.

Drawing inspiration from U-Net and CycleGAN, we propose a novel unsupervised method for low-light image enhancement. Our method employs unpaired image data from both the low-light and normal-light domains for image translation from the low-light to normal-light domain. Our method differs from the original CycleGAN in the following three ways: To begin, we use a U-Net-like structure in the generator to extract features while also introducing new attention based on the original CycleGAN, called feature map attention. The addition of this attention can solve the problems of color cast and exposure while also improving the network’s feature-extraction ability. Second, a style area loss function is added on top of the original loss function to better eliminate the influence of noise. Finally, a new normalization function is introduced to guide the model in recovering more details.

**Figure 2:**
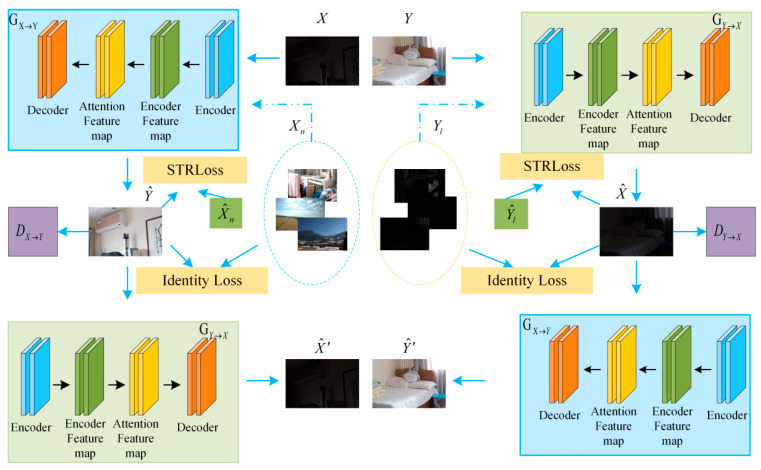
The CGAAN network architecture. X and Y represent the low-light and normal-light images, respectively.$G_{X\to Y}$ and $G_{Y\to X}$ are two generators that represent, respectively, the generation of a normal-light image from a low-light image and the generation of a low-light image from a normal-light image. $D_{X\to Y}$ and $D_{Y\to X}$ are two differentiators. They are the discriminators for images with normal illumination and images with low illumination, respectively.

Figure 2 depicts the structure of our CGAAN. In this section, we first introduce our network’s structure, then the adaptive feature-attention module, and finally, the region loss function and other related loss functions.

### 3.1. Network Architectures

Our CGAAN is made up of two generators and two discriminators, resulting in a cyclic generative adversarial network. As shown in Figure 2, the $G_{X\to Y}$ denotes the generator, which takes the low-light image as input and generates the normal-light image. The figure is represented by $\hat{Y}$. $D_{X\to Y}$ is used to distinguish the normal-light image from the generated normal-light image $\hat{Y}$. $G_{Y\to X}$ indicates that the generated normal-illumination image is used as input to generate the low-illumination image. The cyclic consistency loss is calculated using the original low-light image and the generated low-light image. $D_{Y\to X}$ is used to distinguish the low-light image from the generated low-light image $\hat{X}$. Similarly, $G_{X\to Y}$ takes as input the generated low-light image and produces a normal-light image. The cyclic consistency loss is calculated using both the original and generated normal illumination images. $X_{n}$ and $Y_{l}$ are fed into the generator, and the output is used to calculate the style region loss.

On top of the cycle generative adversarial network, we improve the generator and discriminator. To extract features, we use a U-Net-like structure in the generator. The input image is first processed with a downsampling block, then with a residual block for feature extraction, and the resulting information is fed into the encoded feature map for processing. The attention module then processes it, using regularization to guide the residual blocks and focus the network on more useful information. Finally, an adaptive residual block and an upsampling block comprise the decoder. The goal is to reconstruct the enhanced normal-light image using the feature information that has been collected. The multi-scale information of the various features is fully utilized between the codecs. To the greatest extent possible, we use a global–local discriminator structure to differentiate the generated image from the real image.

### 3.2. Adaptive Feature-Attention Module

**Figure 3:**
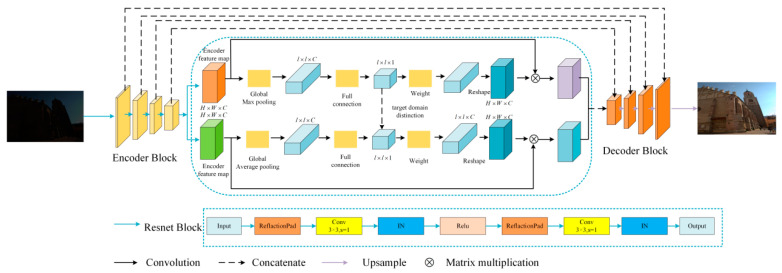
The structure of our generator. It consists of three modules: encoding, adaptive attention, and decoding.

We present an adaptive feature-attention module, the structure of which is shown in Figure 3, to better deal with noise, color deviation, and low-luminance artifacts.

Each pixel in a normally illuminated image is frequently very different from its small neighbors; for example, one pixel may be in normal illumination while the other is in a low-illumination region. The pixels in a neural network’s low-light region are very different from those in the normally illuminated region. However, because of the nature of the convolutional kernel, it typically has a small field of perception and focuses on local information. As a result, after processing, the image’s contextual information is frequently ignored, narrowing the variability between neighboring pixels. As a result, the image is darkly lit and noisy. As a result, we included an attention module with an adaptive feature in the generator. Each channel of the feature information can be customized. Our adaptive feature-attention module adaptively assigns different weights to the feature maps of different channels, focuses more on useful information using global attention, and uses the obtained global information to encode rich contextual information into specific features, better distinguish pixels in the low-light domain from pixels in the normal-light domain, and better eliminate color bias and suppress noise.

The upsampling block, as shown in Figure 3, first upsamples the input low-light image to obtain encoded feature maps before processing it with global max-pooling and global average-pooling to obtain different global information. This feature map is then compressed to one dimension and processed by a class-activation map to better differentiate the low-light domain from the normal-light domain. A channel-weighted attentional feature map is created by reconstructing and weighting the obtained one-dimensional feature map. Finally, the total feature map is computed by multiplying the attention feature map by each original input channel. The entire feature map is fed into the decoding module, which is composed of upsampling blocks, where the features are fused, and the enhanced image is obtained. In general, the attention mechanism we propose compresses the feature map obtained through the pooling operation to one dimension before using the learnable parameters to multiply the corresponding bits of the obtained feature map to obtain the representation. Weights corresponding to feature importance. Based on these weights, the network focuses on critical regions. At the same time, our attention mechanism, when combined with the class-activation map, can adjust the weight or response of features based on the characteristics of low-light and normal-light images, enhancing attention to important features while weakening attention to unimportant features, thereby improving the network’s feature-extraction ability. As a result, the image after enhancement is more realistic.

### 3.3. Loss Functions

In this section, we will discuss the loss functions that we adopt. In this section, adversarial loss, identity-consistency loss, cyclic consistency loss, and style region loss are all introduced in turn.

The loss of adversarial is what brings the generated image closer to the original image. It is described as follows:

(1) $$L_{G_{X\to Y}}=-\log\left(1-D_{X\to Y}\left(G_{X\to Y}(X)\right)\right)$$

Discriminator loss is defined as the following:

(2) $$L_{D_{X\to Y}}=\log\left(D_{X\to Y}\left(X_{n}\right)\right)+\log\left(1-D_{X\to Y}\left(G_{X\to Y}(X)\right)\right)$$

Overexposure and underexposure are frequently encountered during the image-creation process. These phenomena can occur in either relatively bright or very dark areas of low-light images. The degree of improvement is not always clear. To solve this problem, we randomly input several normal-light and low-light images and use identity-consistency loss to enable the network to identify low-light regions and normal-light regions, allowing us to achieve the goal of adaptively enhancing images while avoiding exposure problems. The identity-consistency loss is defined as follows:

(3) $$L_{\mathrm{Identity}}=L_{{\mathrm{Identity}\;}_{l}}+L_{{\mathrm{Identity}\;}_{n}}$$

(4) $$L_{\mathrm{Identity}\;}=\left|Y_{l}-G_{Y\to X}(Y)\right|$$

(5) $$L_{\mathrm{Identity}\;}=\left|X_{n}-G_{X\to Y}(X)\right|$$

We present the cycle-consistency loss inspired by CycleGAN. The cycle-consistency loss ensures that the image generated by the network cycle matches the original input image. Specifically, the cycle-consistency loss directs our network to generate images that match the original input low-light images, ensuring information consistency. The cycle-consistency loss is calculated as follows:

(6) $$L_{cyc_{x}}=\left|X-{\hat{X}}^{\prime }\right|,\;\;\mathrm{where}\;{\hat{X}}^{\prime }=G_{Y\to X}\left(G_{X\to Y}(X)\right)$$

(7) $$L_{\mathrm{cycle}\;}=L_{cyc_{x}}+L_{cyc_{y}}$$

We introduce a style region loss function to help the network distinguish low-light domains from normal-light domains. This loss function can direct the network to produce high-quality images. Given specific images, x∈{X,Y}, $G_{X\to Y}$, and $D_{X\to Y}$, the auxiliary classifiers $\eta_{X}$ and $\eta_{D_{X\to Y}}$ help the network generate higher-quality images by distinguishing between their two domains. The loss function is given as follows:

(8) $$L_{\mathrm{STR}}^{X\to Y}=-\left(E_{x\sim X}\left[\log\left(\eta_{X}(x)\right)\right]+E_{x\sim Y}\left[\log\left(1-\eta_{X}(x)\right)\right]\right)$$

(9) $$L_{\mathrm{STR}}^{D_{X\to Y}}=E_{x∼Y}[(\eta_{D_{X\to Y}}{\left(x\right)}^{2})]+E_{x∼X}[1-\eta_{D_{X\to Y}}{\left(G_{X\to Y}(x)\right)}^{2}]$$

To optimize the final objective, we combine all of the above loss functions. The total loss of the generator is:

(10) $$L_{G}=\lambda_{1}L_{Identity}+\lambda_{2}L_{cycle}+L_{G_{X\to Y}}+L_{CAM}^{X\to Y}$$

The discriminator’s total loss is as the following:

(11) $$L_{D}=L_{D_{X\to Y}}+L_{STR}^{D_{X\to Y}}$$

## 4. Experimental Results and Discussion

In this section, we will first present the specific implementation details of our model. The datasets used for training and testing of all methods are then described, as are the evaluation metrics. Then, we compare our approach to several cutting-edge methods. We used the datasets used by EnlightenGAN for training and testing. For comparison testing, five unpaired datasets (DICM [48], LIME [49], MEF [50], NPE [51], and VV (https://sites.google.com/site/vonikakis/datasets, accessed on 19 May 2023)) were used. Finally, we used an ablation study to validate the plausibility of our network.

### 4.1. Experiment Details

In our experiments, we were able to train our model without using pairs of images. We crop the input images to 256 × 256 pixels in size and train our model for 600 epochs with the Adam optimizer. To reduce model oscillation during training, we create an image buffer to store previously generated images and then use those images to update the discriminator. The initial learning rate is set to 2 × 10^−4^. To improve model convergence, we use a linear decay strategy to dynamically adjust the learning rate. In training, the weights are drawn from a Gaussian distribution N (0, 0.02). The hyperparameters $\beta_{1}$ and $\beta_{2}$ are both set to 0.5 and 0.999. We set $\beta_{1}$ to 0.5 instead of 0.9 because a value of $\beta_{1}$ that is too high can result in unstable network performance. By default, we set $\lambda_{1}$ and $\lambda_{2}$ to 5 and 10. Our model was trained using an RTX A5000 GPU. For the test, we use a 256 × 256 image size.

### 4.2. Datasets and Evaluation Metrics

#### 4.2.1. Datasets

Table 2 shows the datasets that were used in this paper.

We chose the EnlightenGAN dataset for training. The training set in our chosen dataset contains 1016 normal-light images and 914 low-light images, of which 148 are chosen at random for our test set. The resolution of all images was set to 256 × 256 pixels. On the same dataset, we compare the results of supervised and unsupervised methods. We use paired datasets for training the supervised method and unpaired images for training the unsupervised method.

The dataset that was used for testing: The DICM dataset is a low-light image dataset made up of 69 images captured with commercially available digital cameras. The LIME dataset is made up of ten high-quality images. The MEF dataset contains 17 high-quality natural images of indoor and outdoor scenes, natural landscapes, and architectural scenes. The NPE dataset includes 85 real-world images. The VV dataset contains 24 real-world images.

#### 4.2.2. Evaluation Metrics

To ensure a fair comparison, we used three evaluation metrics to assess image quality: peak signal-to-noise ratio (PSNR), structural similarity (SSIM), and natural image quality evaluation (NIQE).

Peak Signal-to-Noise Ratio (PSNR). The peak signal-to-noise ratio is a metric for evaluating images. The higher the value, the higher the quality of the image. It is described as follows:

(12) $$\mathrm{PSNR}=10\times \log_{10}\left(\frac{MAX_{I}^{2}}{MSE}\right)=20\times \log_{10}\left(\frac{MAX_{I}}{\sqrt{MSE}}\right)$$

where the maximum value of the image color is represented by $MAX_{I}$, and 8-bit sampling points are represented by 255.

Structural Similarity (SSIM). Structural similarity is a full-reference image quality evaluation index that calculates image similarity based on three factors: brightness, contrast, and structure. The value range is 0–1, with larger values indicating less distortion. It is described as follows:

(13) $$\mathrm{SSIM}(x,y)=\frac{\left(2\mu_{x}\mu_{y}+c_{1}\right)\left(2\sigma_{xy}+c_{2}\right)}{\left(\mu_{x}^{2}+\mu_{y}^{2}+c_{1}\right)\left(\sigma_{x}^{2}+\sigma_{y}^{2}+c_{2}\right)}$$

where $\mu_{x}$ represents the mean of x, $\mu_{y}$ represents the mean of y, $\sigma_{x}^{2}$ represents the variance of x, $\sigma_{y}^{2}$ represents the variance of y, and $\sigma_{xy}$ represents the covariance of x and y. $c_{1}={\left(k_{1}L\right)}^{2}$ and $c_{2}={\left(k_{2}L\right)}^{2}$ are stability coefficients, and L is the dynamic range of pixel values. $k_{1}=0.01$, $k_{2}=0.03$.

Natural Image Quality Evaluation (NIQE). Natural image quality assessment is an objective evaluation index that extracts features from natural images and fits them into a multivariate Gaussian model. Lower values indicate higher image quality.

### 4.3. Comparison with State-of-the-Art Methods

We compare our method to the most recent methods. DALE [52], DRBN [53], DSLR [54], EnlightenGAN [17], RUAS [55], Zero-DCE [30], SGZ [56], and SCI [57] are among them. The metric results are displayed later.

#### 4.3.1. Qualitative Comparisons

**Figure 4:**
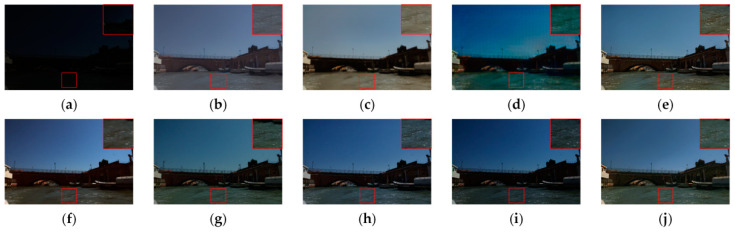
Visual comparison with other advanced methods on the EnlightenGAN dataset. The image’s key details are indicated in red boxes. (**a**) Input, (**b**) DALE, (**c**) DRBN, (**d**) DSLR, (**e**) EnlightenGAN, (**f**) RUAS, (**g**) Zero-DCE, (**h**) SGZ, (**i**) SCI, (**j**) ours.

**Figure 5:**
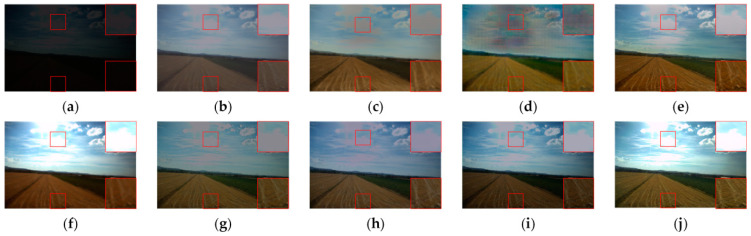
Visual comparison with other advanced methods on the EnlightenGAN dataset. The image’s key details are indicated in red boxes. (**a**) Input, (**b**) DALE, (**c**) DRBN, (**d**) DSLR, (**e**) EnlightenGAN, (**f**) RUAS, (**g**) Zero-DCE, (**h**) SGZ, (**i**) SCI, (**j**) ours.

**Figure 6:**
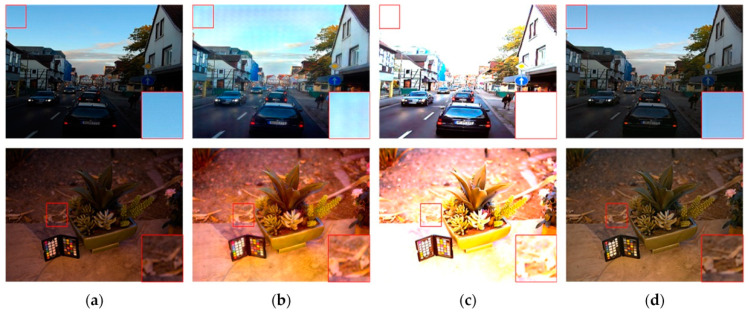
Visual comparison with other methods on the LIME dataset. The image’s key details are indicated in red boxes. (**a**) Input, (**b**) DSLR, (**c**) RUAS, (**d**) ours.

**Figure 7:**
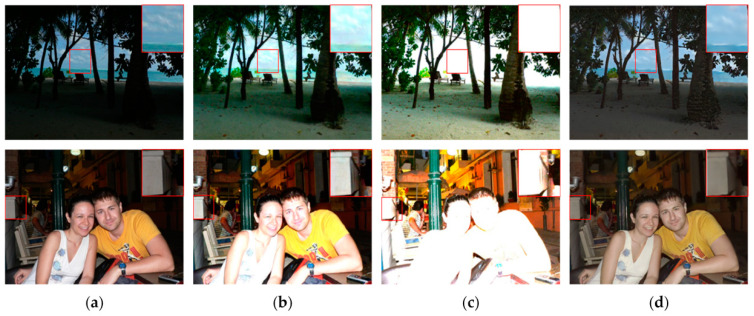
Visual comparison with other methods on the VV dataset. The image’s key details are indicated in red boxes. (**a**) Input, (**b**) DSLR, (**c**) RUAS, (**d**) ours.

We began by comparing the visual quality of our method to that of other advanced methods. Figure 4 and Figure 5 depict the specific outcomes. As shown in the figures, all of the methods in the comparison improve the images to some extent on the test dataset we used, but they still lack brightness when compared to our method. Figure 4 shows that the boxed and labeled parts improve the image’s brightness but leave it with very low color saturation. After DSLR enhancement, there are some areas of color imbalance. Our method, on the other hand, exhibits no color bias and no overexposure. Figure 5 shows that there are some areas of artifacts, overexposure, and overall poor color saturation in the enhanced images produced by the comparison method. In contrast, our method produces almost no artifacts and has high color saturation. We chose the LIME and VV datasets for testing in order to further validate the performance of our method. Figure 6 and Figure 7 show the specific results. We can see from the first row of images in Figure 6 that the DSLR method produces color patches, and the RUAS method overexposes. In contrast, our method avoids the aforementioned issues. Our method preserves more color detail in the second row of images while enhancing and avoiding overexposure. The DSLR method produces a color bias in the first row of Figure 7, while the RUAS method overexposes in the marked areas. Our method preserves color detail without overexposing it. As the results show, our method does a good job of avoiding artifacts and overexposure.

#### 4.3.2. Quantitative Comparisons

We quantitatively compare our method to others. The colors red, green, and blue represent the top three best results for the corresponding metrics. The PSNR and SSIM metrics of various methods on the EnlightenGAN dataset are shown in Table 3. Higher PSNR values indicate better image quality, while higher SSIM values indicate greater similarity between images and better image quality. As shown in Table 3, our method achieves the highest PSNR value while also having the second-best SSIM value. Unlike previous attention modules that focused solely on feature extraction without taking into account semantic information or global–local relationships, our feature-attention module focuses on the important regions between feature maps, taking both semantic and global information into account, resulting in a more realistic image. As a result, our method has the highest PSNR and the second-best SSIM value. The NIQE metric values of the different methods on the five datasets are listed in Table 4. The lower the NIQE value, the more natural the image. Our method achieves the best results on the NPE and VV datasets and the third-best results on the average of the five datasets, as shown in Table 4.

In addition to the above evaluation metrics, the algorithm’s running time should be considered. The extremely short running time can significantly improve the algorithm’s efficiency and ensure a thorough evaluation of the algorithm.

**Figure 8:**
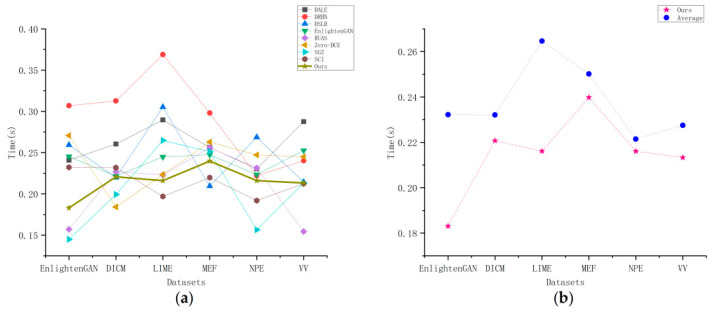
A comparison of the running times of the methods used on different datasets. (**a**) Comparison of our method’s running time with other state-of-the-art methods, (**b**) Comparison of our method’s average running time with other state-of-the-art methods.

Table 5 compares the running times of various algorithms on the datasets used. As shown in Table 5, our method outperforms the majority of the algorithms in terms of running time. The SGZ algorithm has the shortest running time on the EnlightenGAN dataset because it uses depthwise separable convolutions to estimate low-light images at the pixel level, but it does not perform well on other datasets. This could be due to differences in dataset acquisition and image quality. The Zero-DCE algorithm estimates the task using a specific curve and, to some extent, reduces the running time. Because of the use of a self-calibration module, the SCI method has short run times. By processing images on the basis of Retinex, the RUAS method can also reduce running time. The DSLR method restores image features using the Laplacian pyramid structure and has good performance. Our method, on the other hand, outperforms the majority of the comparison algorithms. Our method employs new attention to quickly capture useful information in images and employs a U-Net-like structure to improve the network’s feature-extraction ability, resulting in a relatively short running time. Figure 8 depicts the running time, and it can be seen that our method outperforms the majority of the comparison methods. In Figure 8b, we compared our method’s running time to the average running time of other methods. It can be seen that our method outperforms the average and has good performance.

**Figure 9:**
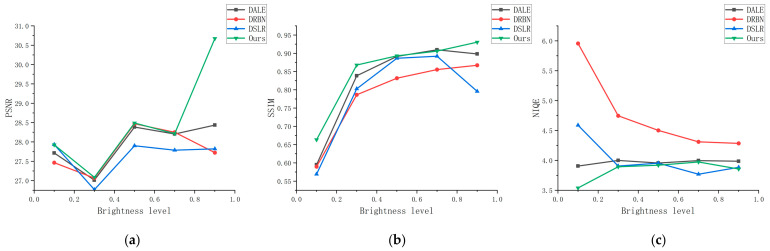
On images of varying brightness, the evaluation metrics of our method were compared to those of other cutting-edge methods. (**a**) PSNR, (**b**) SSIM, (**c**) NIQE.

A good algorithm should have good generalization. As a result, in order to test the generalization of our method, we tested our algorithm in various lighting conditions. To obtain images with varying brightness, we divide the brightness level into five levels for processing, with brightness values ranging from 0.1 to 0.9 with an interval of 0.2. The highest brightness is represented by 1.0. We chose DALE, DRBN, and DSLR to compare with our method in order to show the results more intuitively. Figure 9 displays the test results.

Figure 9 shows that the index values of images processed by our method are generally higher than those of other methods. This is because our new attention solves potential color bias and exposure issues, the style region loss removes noise to some extent, and the new normalization function retains more semantic information. The test results demonstrate our method’s generalizability even further.

The FLOPs metric comparison between our method and other methods is shown in Table 6. Table 6 shows that our method has a higher FLOPs metric value than the others and requires more computing resources. This is due to the fact that the addition of our individual modules increases the model’s computational resources. The comparison of the aforementioned indicators demonstrates that our method has some limitations. Our method’s limitation is that it requires more computing resources and takes longer to run. To reduce the computing resources occupied by the model, we will consider introducing model pruning and using lightweight models in the future.

### 4.4. Ablation Experiment

In this section, we conduct ablation experiments to validate the efficacy of our method. The adaptive feature-attention module and the style region loss function are included.

The significance of each component. Table 7 shows the quantification results. As shown in Table 7, the values of the PSNR and SSIM metrics decrease significantly when each component is removed when compared to the complete method. As shown in the table, the impact on the metrics is greatest after the loss of a style region. This is because style region loss can direct the network to pay more attention to the relationship between different styles, resulting in high-quality images. The normalization function has the least impact on the metric values, but its absence still causes a decrease in the metric values. The quantitative results in the table demonstrate the significance of each proposed module.

**Figure 10:**
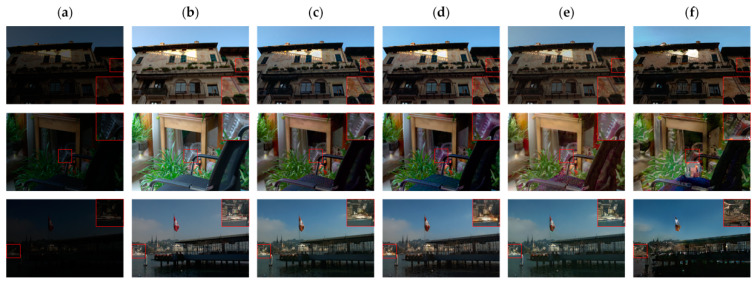
A visual comparison of our ablation technique. Our approach works well. (**a**) Input, from left to right, (**b**) standard, (**c**) our approach, (**d**) insufficient adaptive attention, (**e**) there will be no loss of style across regions, (**f**) no function for normalization.

Figure 10 shows the qualitative comparison results to further demonstrate the impact of our adaptive feature-attention module, stylized region loss, and normalization function on the experimental results. Figure 10 shows how the feature-attention module can extract more useful feature information to reduce noise and color bias, while the region loss and normalization function can guide the network to focus on differences between regions and solve the exposure problem. The findings in Figure 10 support the significance of our module.

We conducted an ablation study on the loss term to further demonstrate the effectiveness of our method. Table 8 shows the specific results. As shown in Table 8, the performance results of our proposed loss are the best. The absence of identity-consistency loss and cycle-consistency loss has a greater impact on the results because identity-consistency loss ensures the integrity of semantic information before and after enhancement, and cycle-consistency loss ensures the consistency of background information before and after enhancement. Furthermore, when compared to Table 3, the absence of any loss item will have an effect on the results. This clearly demonstrates the efficacy of our method.

Our method performs well on multiple indicators, which may be useful for future computer vision tasks. The majority of current target detection tasks are based on images with normal lighting, and there is insufficient research on target detection in low-light conditions. Potential noise and insufficient contrast in low-light conditions degrade the performance of low-light object detection and make the task more difficult. Specifically, our method reduces the impact of potential noise while improving image quality, effectively improving object detection performance in low-light conditions.

### 4.5. Application

**Figure 11:**
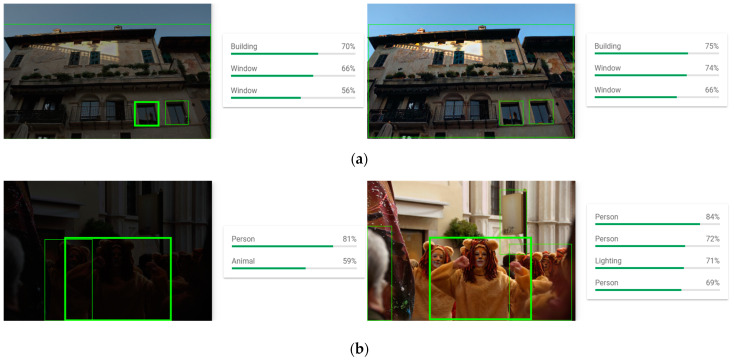
EnlightenGAN dataset results from the Google Cloud Vision API. The detected targets are denoted by the green box. (**a**) The raw results of low-light image detection; (**b**) the enhanced image’s detection results.

We use the Google Vision API to test our output results in order to further demonstrate the feasibility of our proposed method for low-light object detection. Figure 11 depicts the outcomes.

Figure 11a shows that, when compared to the original low-light image, our method’s image recognition confidence after enhancement is higher, and the recognition effect is better. Figure 11b shows that there are detection errors and missed detections in the original low-light image. Street lights, for example, are not detected in the original low-light image. Our method, on the other hand, solves these issues and produces good detection results. The preceding findings support the significance of our method and its applicability to computer vision object-detection tasks.

## 5. Future Work

According to the literature, low-light image enhancement still faces many challenges, and there are numerous directions for future research.

(1) In conjunction with some specific network structures. Using an appropriate network structure can significantly improve the quality of the enhanced image. Although the majority of the previous methods have been improved based on the U-Net network structure, this does not guarantee that they can be applied to all low-light image enhancement situations. Considering low-light images with low contrast and small pixel values, a suitable network structure for enhancement must be chosen;
(2) Integrate Semantic Data. Semantic information includes image features such as color, which allows the network to distinguish regions of different brightness in the image, which is extremely useful for detail restoration. As a result, combining the benefits of semantic information with semantic information will be a hot research topic in the future;
(3) Given the complexity of low-light image enhancement tasks, investigating how to adaptively adjust the enhancement degree based on user input and how to combine it with sensors is also a promising future research direction.

## 6. Conclusions

CGAAN (cyclic generative attention-adversarial network), an unsupervised low-light image-enhancement network, is proposed in this paper. Our approach differs from existing methods in that we use stylized region loss, which allows the network to pay more attention to the differences between low-light and normal-light images and thus guides the network to generate higher-quality images. Furthermore, the new normalization function we introduce can retain more semantic information, which simplifies the subsequent tasks. It is worth noting that our attention module differs from previous attention modules in that it can guide the generator to distinguish low-light images from normal-light images while transitioning in important regions, thereby ensuring image quality. Specifically, the newly introduced feature-attention module in our network focuses on more useful regions in the input low-light image to solve the noise problem, exposure problem, and color bias problem. Meanwhile, the newly introduced region loss function causes the network to pay more attention to differences between regions and improves the network’s ability to recover details, bringing the generated image closer to the true image. Furthermore, the newly introduced adaptive normalization function can extract stylistic features more accurately, resulting in higher image quality. We ran qualitative and quantitative experiments on a variety of low-light datasets, and the results show that our method can improve image quality, which can aid in subsequent tasks.

## Figures and Tables

**Table 2:** Comparison of the datasets used in this article.

Dataset	Scale	Synthetic/Real	Paired/Unpaired
EnlightenGAN	914 low-light images, 1016 normal-light images	Synthetic	Unpaired
DICM	69 images	Real	Unpaired
LIME	10 images	Real	Unpaired
MEF	17 images	Real	Unpaired
NPE	85 images	Real	Unpaired
VV	24 images	Real	Unpaired

**Table 3:** PSNR and SSIM comparisons using different methods on the EnlightenGAN dataset (The higher the PSNR value, the better the image quality. The higher the SSIM value, the better the image quality. Red, green, and blue represent the top-three metric values, respectively).

Method	PSNR	SSIM
DALE	27.1563	0.7770
DRBN	29.3280	0.6661
DSLR	28.9918	0.9536
EnlightenGAN	27.5032	0.8090
RUAS	30.8243	0.9242
Zero-DCE	31.1470	0.9097
SGZ	29.8204	0.8213
SCI	31.4300	0.9501
Ours	31.5482	0.9504

**Table 4:** NIQE on five different data sets (Higher image quality is indicated by lower NIQE values. Red, green, and blue represent the top-three indicator values, respectively).

Method	DICM	LIME	MEF	NPE	VV	Average
DALE	3.4665	2.9993	3.7530	3.5869	3.3174	3.4246
DRBN	3.8223	3.6287	4.5318	4.6310	4.1312	4.4190
DSLR	3.2065	3.8683	3.4878	3.6515	3.5184	3.5465
EnlightenGAN	3.4095	2.9206	2.8039	3.5371	3.6619	3.2666
RUAS	3.1667	3.3964	4.0510	3.8972	6.1626	4.1384
Zero-DCE	2.6201	3.1936	3.5447	3.1849	3.4984	3.2083
SGZ	2.6263	3.5883	3.8319	3.8319	3.6173	3.4991
SCI	3.1113	3.6580	3.1131	3.2434	4.2304	3.4712
Ours	3.2299	3.9004	3.8583	2.8435	3.2824	3.4229

**Table 5:** Compares the runtimes (in seconds) of the datasets used in the comparison methods (red, green, and blue represent the top-three indicator values, respectively).

Method	EnlightenGAN	DICM	LIME	MEF	NPE	VV
DALE	0.2409	0.2605	0.2898	0.2562	0.2304	0.2876
DRBN	0.3069	0.3126	0.3689	0.2980	0.2220	0.2403
DSLR	0.2594	0.2199	0.3053	0.2095	0.2686	0.2143
EnlightenGAN	0.2453	0.2216	0.2449	0.2475	0.2234	0.2526
RUAS	0.1570	0.2269	0.2230	0.2559	0.2314	0.1544
Zero-DCE	0.2708	0.1842	0.2235	0.2629	0.2472	0.2450
SGZ	0.1450	0.1993	0.2649	0.2513	0.1565	0.2133
SCI	0.2322	0.2318	0.1970	0.2198	0.1919	0.2125
Ours	0.1831	0.2207	0.2161	0.2398	0.2161	0.2133

**Table 6:** Compares our method’s FLOPs (G) to other methods.

Method	FLOPs
DALE	3017.28256
DRBN	339.30451
DSLR	188.00941
EnlightenGAN	526.23416
RUAS	6.85140
Zero-DCE	166.09444
SGZ	5.35141
SCI	3155.95162
Ours	2300.29894

**Table 7:** PSNR and SSIM index values for ablation experiments were compared. The symbol × indicates that the corresponding module is not in use, while the symbol √ indicates that it is in use.

Adaptive Feature Attention	Loss of Style Region	Normalization Function	PSNR	SSIM
×	√	√	30.2435	0.9417
√	×	√	30.1493	0.8751
√	√	×	30.8110	0.9485
√	√	√	31.5482	0.9504

**Table 8:** Loss-term ablation experiments. The symbol × indicates that the corresponding module is not in use, while the symbol √ indicates that it is in use.

Loss of Identity Consistency	Cycle-Consistency Loss	Style Region Loss	PSNR	SSIM
×	√	√	27.8482	0.53951
√	×	√	28.7930	0.7182
√	√	×	30.1493	0.8451
√	√	√	31.5482	0.9504

## Data Availability

Not applicable.
